# App-Controlled Treatment Monitoring and Support for Head and Neck Cancer Patients (APCOT): Protocol for a Prospective Randomized Controlled Trial

**DOI:** 10.2196/21693

**Published:** 2020-12-09

**Authors:** Tanja Sprave, Daniela Zöller, Raluca Stoian, Alexander Rühle, Tobias Kalckreuth, Erik Haehl, Harald Fahrner, Harald Binder, Anca-Ligia Grosu, Felix Heinemann, Nils Henrik Nicolay

**Affiliations:** 1 Department of Radiation Oncology Faculty of Medicine and University Medical Center Freiburg University of Freiburg Freiburg Germany; 2 German Cancer Consortium (DKTK) Partner Site Freiburg German Cancer Research Center Heidelberg Germany; 3 Institute of Medical Biometry and Statistics Faculty of Medicine Freiburg Germany

**Keywords:** mHealth, head and neck cancer, HNSCC, radiotherapy, mobile app, quality of life, patient-reported outcome measures

## Abstract

**Background:**

Head and neck cancers (HNCs) are among the most common malignancies, which often require multimodal treatment that includes radiation therapy and chemotherapy. Patients with HNC have a high burden of symptoms due to both the damaging effects of the tumor and the aggressive multimodal treatment. Close symptom monitoring over the course of the disease may help to identify patients in need of medical interventions.

**Objective:**

This APCOT (App-Controlled Treatment Monitoring and Support for Head and Neck Cancer Patients) trial is designed to assess the feasibility of monitoring HNC patients during the course of (chemo)radiation therapy daily using a mobile app. Additionally, symptom patterns, patient satisfaction, and quality of life will be measured in app-monitored patients in comparison to a patient cohort receiving standard-of-care physician appointments, and health economy aspects of app monitoring will be analyzed.

**Methods:**

This prospective randomized single-center trial will evaluate the feasibility of integrating electronic patient-reported outcome measures (ePROMs) into the treatment workflow of HNC patients. Patients undergoing definitive or adjuvant (chemo)radiation therapy as part of their HNC treatment at the Department of Radiation Oncology, University Medical Center Freiburg (Freiburg, Germany) will receive weekly physician appointments and additional appointments as requested to monitor and potentially treat symptoms during the course of treatment. Patients in the experimental arm will additionally be monitored daily using a dedicated app regarding their disease- and treatment-related symptoms, quality of life, and need for personal physician appointments. The feasibility of ePROM monitoring will be tested as the primary endpoint and will be defined if ≥80% of enrolled patients have answered ≥80% of their daily app-based questions. Quality of life will be assessed using the validated European Organisation for Research and Treatment of Cancer questionnaires, and patient satisfaction will be measured by the validated Patient Satisfaction Questionnaire Short Form at the initiation, in the middle, and at completion of radiation therapy, as well as at follow-up examinations. Additionally, the number and duration of physician appointments during the course of radiation therapy will be quantified for both ePROM-monitored and standard-of-care patients.

**Results:**

This trial will enroll 100 patients who will be randomized (1:1) between the experimental arm with ePROM monitoring and the control arm with standard patient care. Recruitment will take 18 months, and trial completion is planned at 24 months after enrollment of the last patient.

**Conclusions:**

This trial will establish the feasibility of close ePROM monitoring of HNC patients undergoing (chemo)radiation therapy. The results can form the basis for further trials investigating potential clinical benefits of detailed symptom monitoring and patient-centered care in HNC patients regarding oncologic outcomes and quality of life.

**Trial Registration:**

German Clinical Trials Register DRKS00020491; https://www.drks.de/drks_web/navigate.do?navigationId=trial.HTML&TRIAL_ID=DRKS00020491

**International Registered Report Identifier (IRRID):**

PRR1-10.2196/21693

## Introduction

Head and neck carcinomas (HNCs) are among the most common malignancies worldwide with more than 600,000 new diagnoses and 400,000 deaths annually [1]. Disease-associated morbidities are widespread in HNC patients and can affect the ability to breathe or swallow, often requiring life-saving placements of tracheostomy or gastrostomy tubes [2-4]. For nonmetastatic HNCs, multimodal treatment comprises surgery and radiation therapy, and locally advanced cancers often require a combination of radiation therapy and concomitant chemotherapy [5,6]. The treatment itself can result in severe acute and long-lasting toxicities that may significantly impact patients’ quality of life and require medical interventions [7].

Usually, treatment-related acute toxicities are assessed several times during the course of (chemo)radiation therapy and in increasing intervals during the follow-up period by medical professionals. However, there are several downsides to physician-assessed outcome measures for HNC patients. There are increasing gaps between appointments, during which no assessment is possible and patients may get lost to follow up; additionally, patients may not report the entirety of their symptoms during timed interactions with their health care provider [8,9]. In this respect, mobile apps may help to bridge the gap between appointments, and may provide a low-threshold tool to regularly and frequently report disease-related and treatment-related symptoms and outcomes. Previous surveys have demonstrated high acceptance levels for the usage of patient monitoring apps both for health care providers and cancer patients [10,11]. Additionally, two randomized controlled trials have been published that demonstrated a survival benefit of telemonitoring for patients with nonsmall cell lung cancers and metastatic cancers compared to the standard of care, likely due to an earlier initiation of salvage treatments in cases of recurrence symptoms [12,13].

HNC patients may especially benefit from app-based electronic patient-reported outcome measures (ePROM) monitoring, as frequent symptom control may not only help to detect recurrences earlier but could also help to assess the dynamics of treatment-related toxicities, thereby enabling faster medical interventions. In this respect, both patient outcomes and quality of life may potentially be improved. However, the compliance and acceptance levels of HNC patients toward an app-based ePROM monitoring system remain to be demonstrated. The aim of this trial, APCOT (App-Controlled Treatment Monitoring and Support for Head and Neck Cancer Patients), is to assess the feasibility of ePROM monitoring and support for HNC patients, as well as to measure global and disease-related quality of life, patient satisfaction, and economic aspects.

## Methods

### Study Design

This trial is designed as a single-center prospective randomized controlled trial and will be carried out at the Department of Radiation Oncology, University of Freiburg Medical Center in Freiburg, Germany. Patients with histologically proven HNCs scheduled for (chemo)radiation therapy will receive weekly physician appointments and additional appointments if medically indicated in both trial arms to monitor and potentially treat symptoms occurring during the course of treatment (standard of care). Patients in the experimental arm will additionally receive daily ePROM monitoring during (chemo)radiation. Patient-reported outcomes regarding symptom control as well as general and disease-specific quality of life will be monitored daily by a mobile app in the experimental arm that is provided to the patients on a tablet computer. All patients in the experimental arm will have the ability to request a physician appointment if necessary on a daily basis via the app. The frequency and total duration of consultations will be quantified, and the necessity and duration of inpatient care will be assessed if necessary during the course of treatment. Routine blood test results and clinical data from the patient files, including physician-reported outcomes, will be analyzed to find potential correlations with the ePROMs and to assess potential predictive factors necessitating intensified medical care.

### Patient Recruitment

All patients with HNCs are screened for their eligibility to participate in this trial by a study nurse prior to the initiation of (chemo)radiation therapy; the treating physician then informs potential patients about the details of the trial and answers potential questions. If (chemo)radiation therapy is medically warranted and patients provide written informed consent to participate, they are randomized between the standard-of-care monitoring and ePROM monitoring arms during the course of treatment. Block randomization with a variable block length will be used to allocate patients to the study arms.

**Figure 1:**
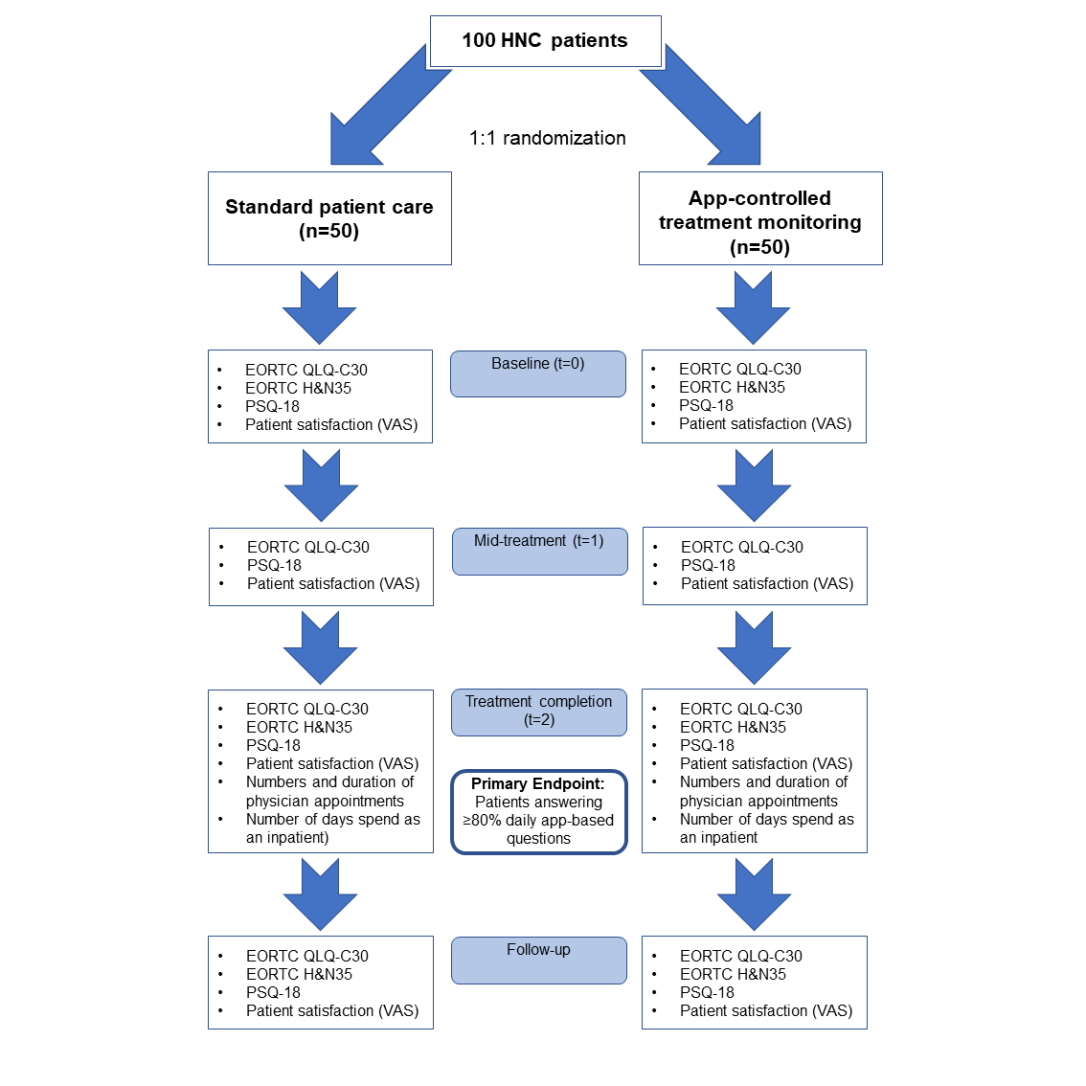
Flow chart outlining the study design and the timeline for the individual trial endpoints. HNC: head and neck cancer; VAS: visual analog scale; EORTC: European Organisation for Research and Treatment of Cancer; PSQ-18: Patient Satisfaction Questionnaire Short Form.

According to institutional patient statistics, approximately 300 eligible patients are treated annually in our department. The recruitment period is set at 18 months to recruit 50 patients to each study arm, assuming a participation rate of 20%. The study duration for each patient varies according to the treatment concept, and the average time from enrollment in the trial to the first follow up ranges from 12 to 14 weeks. Figure 1 provides a detailed timeline for the individual trial steps and assessments. The SPIRIT (Standard Protocol Items: Recommendations for Interventional Trials) flowchart is presented in Multimedia Appendix 1.

### Inclusion and Exclusion Criteria

To participate in this trial, patients must fulfill the following inclusion criteria: (1) histologically confirmed tumor in the head and neck region, (2) indication for chemoradiation or radiation therapy, (3) aged ≥18 years, (4) Karnofsky performance score ≥50%, and (5) provision of written informed consent.

The following criteria will exclude patients from participating in this trial: (1) significant neurological or psychiatric diseases and (2) inability to give informed consent.

### ePROM Monitoring During Treatment and Support

Patients that are randomized to the experimental arm will be provided with a mobile app on a tablet computer prior to each radiation treatment fraction and will be asked to answer 7-8 questions. The app will be provided by ONCARE GmbH (Munich, Germany) and will be run on an Android system. An introductory session with a study nurse will be scheduled for each patient in the experimental arm to familiarize them with the tablet and the app. If requested, this introductory session can be repeated once. The answers will be collected and documented as patient-reported outcomes. Questions will be taken from the validated H&N35 quality of life questionnaire developed by the European Organisation for Research and Treatment of Cancer (EORTC). The questions cover overall and treatment-related well-being as well as specific symptoms, and the 35 questions of the EORTC module will be rotated so that each patient is presented with each question once weekly. As the respective questionnaires have been validated in multiple languages, the monitoring app will be multilingual. After completing the daily EORTC questions, patients will be asked about their need to consult a physician on each treatment day. If they require a physician appointment, the radiation technologist is informed by an automatic notification system prior to applying the daily treatment and will schedule a consultation with the treating physician on that day. Each patient in the experimental study arm will be seen by the treating physician for routine checkups weekly in addition to the visits requested through the app. The total number and duration of all physician appointments will be measured and documented.

Patients in the standard-of-care arm will receive routine weekly physician appointments during the course of treatment and can request additional consultations through the treating radiation technologist as per institutional standards. Patients in both study arms will also receive weekly blood tests. As the standard-of-care physician appointments are also part of the experimental arm, the mobile therapy monitoring provides an additional individualized method of care during (chemo)radiation therapy.

**Figure 2:**
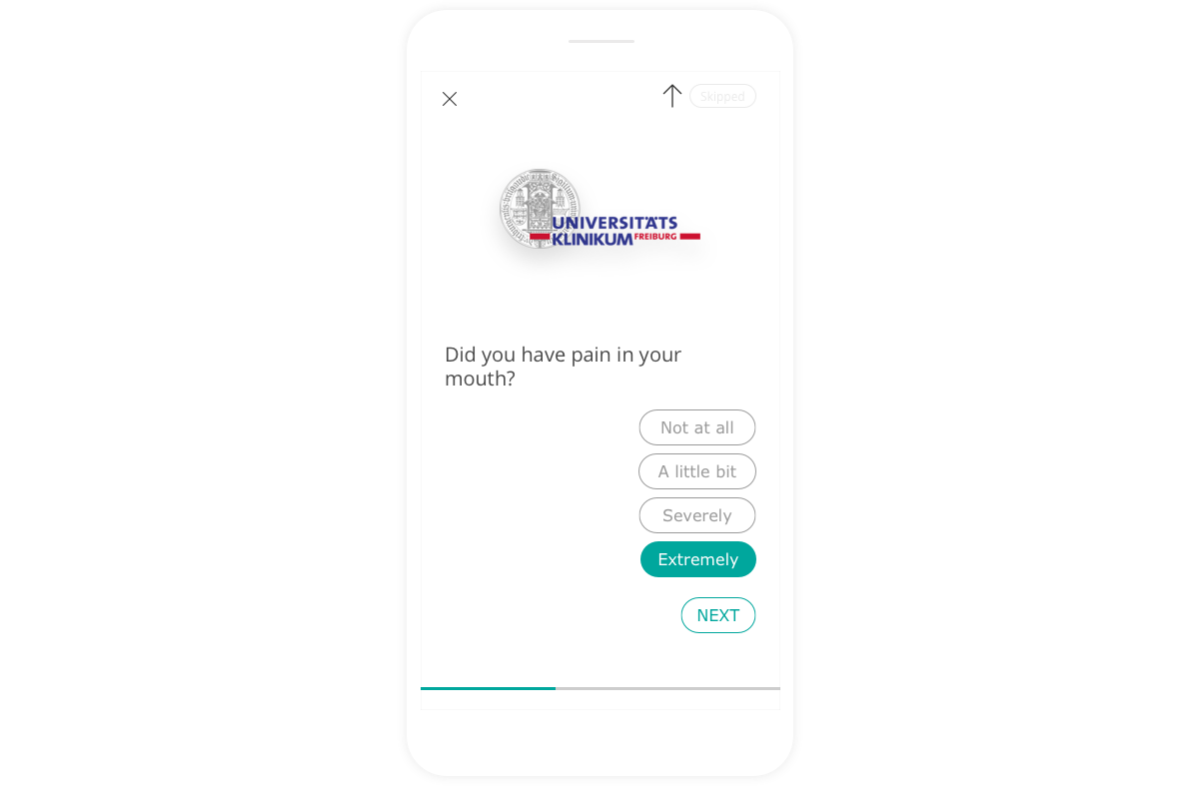
Sample screenshot of the mobile app used for patient monitoring during radiation therapy.

A sample screenshot of the mobile app is shown in Figure 2.

### Primary and Secondary Endpoints

This trial aims to test the feasibility of an ePROM-based treatment monitoring and support system for HNC patients as the primary endpoint. The trial will measure the percentage of patients answering ≥80% of ePROM questionnaires during the course of (chemo)radiation therapy, and the number of answered questions for each treatment day will be documented for every patient to assess feasibility. Feasibility in this trial is defined based on clinical and logistical considerations. We assume that due to logistic constraints unrelated to this trial, patients will not receive ePROM questionnaires for a certain percentage of treatment fractions (eg, due to personnel changes, technical outages, postponement of treatment fractions). Based on clinical experience from preliminary tests, we assume that the proportion of irregularities during routine patient treatment will amount to approximately 20%.

The following secondary endpoints will also be measured: patient satisfaction, health-related quality of life, number and duration of physician appointments, number of days spent as an inpatient for treatment-related toxicities, and cost aspects. Patient satisfaction will be measured by a 10-step visual analog scale and the validated Patient Satisfaction Questionnaire Short Form (PSQ-18) questionnaire that tests 18 items in 7 subscales using a Likert scale ranging from 1 to 5. A total sum score will be calculated from the PSQ-18 questionnaire and will be transformed to a 5-point scale. Health-related quality of life for patients in both study arms will be measured using the validated EORTC QLQ-C30 questionnaire.

Patients will be asked to answer questionnaires before the initiation of (chemo)radiation therapy, in the middle (usually week 4), and upon completion of treatment (Figure 1). Additionally, patients will be presented with the questionnaires at follow-up visits.

The number of all physician appointments will be documented, and the duration of individual appointments will be measured to estimate cost factors for ePROM monitoring during treatment. The beginning and completion of physician appointments are routinely marked by status flags by the physician within the hospital’s radiation oncology information system (MOSAIQ, Elekta, Stockholm, Sweden), and the duration of each individual consultation can therefore be extracted from the system. A randomized design was chosen for this trial to assess the completeness of acquired data between both arms and to compare the two groups regarding the secondary endpoints.

### Statistical Analysis

The sample size was calculated based on the primary endpoint using the exact binomial test. For this trial, we defined feasibility as the ability of ≥80% of trial patients to answer ≥80% of mobile ePROM questions during the course of (chemo)radiation therapy. With an assumed percentage of 89% for the primary endpoint, a sample size of 50 patients in the experimental arm provides a power of 80% (1 – β) to a one-sided significance level of 5% (α). Power calculations were carried out using G*Power software version 3.1.9.2 (University of Düsseldorf, Germany).

Descriptive statistics will be used to analyze the secondary endpoints, including mean (SD) as well as median (IQR) for continuous variables, and absolute and relative numbers for categorical variables. Multivariate regression models will be employed to assess potential correlations between clinical parameters and data collected for the primary and secondary endpoints. The control group will be included in the analysis, and the randomization of each patient will be considered as a variable. Sum scores will be derived from the quality of life and patient satisfaction questionnaires, and will be compared between study arms using Mann-Whitney *U* tests.

To investigate the association between patient-reported outcomes and the necessity for inpatient treatment, a chi-square test will be employed.

### Informed Consent and Ethics Approval

Participation in this trial is voluntary, and all patients qualifying for enrollment will be informed by the treating physician about the aims, risks, and benefits of the trial, and will be provided with written information and the consent form. Both documents conform to the standards of the International Conference on Harmonization-Good Clinical Practice. All patients will be given appropriate time to consider participation before providing informed consent in writing, including the date and time of signature. Consent forms will be countersigned by the physician. In cases of incapability to sign the consent form, oral informed consent will be confirmed by a witness signature. All patients can decline to participate in this trial and can withdraw consent at any time during the trial without penalty. Patient treatment will not be affected by the participation or withdrawal from this trial. All trial data will be saved on a dedicated trial server, and only the staff directly involved in the trial will have access to the files. All trial data will be saved for 10 years as per national guidelines and regulations. Data will not be shared with third parties.

The trial was approved by the Independent Ethics Committee of the University of Freiburg on November 26, 2019 (reference number 87/19).

## Results

Recruitment for this trial started in September 2020, and patient enrollment is planned to be completed in February 2022. The results obtained from this trial will be published in a peer-reviewed journal within 12 months of completion of the trial; publication of the data will be independent of the trial outcome. The principal investigator will be responsible for the preparation of any publications resulting from this trial and will assign the first and last authorships.

## Discussion

### Study Rationale

The widespread availability of mobile electronic devices, and resulting possibilities to interact with and support cancer patients via electronic means enable health care providers to collect disease-related data and notify patients about necessary appointments or test results via dedicated apps. Patients with locally advanced HNCs may benefit from ePROM monitoring during their treatment both regarding the control and recurrence of disease symptoms, and for longitudinal assessment of the often significant treatment-related toxicities. In this respect, HNC patients may be scheduled for earlier medical interventions if necessary, thus improving both oncological outcomes and quality of life. This prospective randomized trial conducted at University of Freiburg Medical Center will investigate the feasibility of monitoring ePROMs of HNC patients via a mobile app during the course of (chemo)radiation therapy, and will measure resulting general and disease-related quality of life, patient satisfaction, and implications regarding health economics.

### Limitations and Future Perspectives

This trial involves a mobile app used by patients via an investigator-provided tablet computer, and the acquired data will be integrated into the institutional clinical trial databases according to the required data protection and data security regulations. It is conceivable that a dedicated app on patients’ private devices would have logistic advantages and enable patients to report outcome parameters independently from the treatment time and from outside as well. However, we felt that utilizing patient-owned devices for reporting may introduce a selection bias, as this approach would exclude patients who do not own a mobile device, especially elderly patients [14]. By using investigator-provided tablet computers, all HNC patients, irrespective of age or technical abilities, can be included in this trial. Additionally, avoidance of data transmission outside the hospital network complies with current national data protection standards.

As each cycle of ePROM collection is carried out over 5 days, it is possible that symptom reporting may be delayed for up to 4 days before items are repeated. However, in case of new symptoms, all patients in the experimental arm have the ability to request physician appointments daily via the app if deemed necessary. The frequency of ePROM assessment corresponds well with current national and international standards of weekly physician appointments during radiotherapy.

Once feasibility has been demonstrated for HNC patients using institutional devices, the range of the app will be expanded in a future trial to patients’ personal devices so as to uncouple reporting from the hospital visits.

To date, there is no consensus on how feasibility for app-based treatment monitoring is defined and can be tested. We therefore chose a feasibility endpoint for this trial based on clinical experience. Two previously published large surveys suggest that more than 75% of patients would be willing to report ePROMs as part of treatment monitoring during radiation therapy [10,15]. Therefore, patient adherence of ≥80% is within the estimations of these surveys and seems to be a realistic assumption to prove feasibility.

Beyond demonstrating that ePROM monitoring of HNC patients during radiotherapy is feasible, this trial will provide systematic and specific patient-reported outcomes for a distinct patient population. These data may serve as a template to develop thresholds and patterns for high-risk constellations that require medical interventions and will therefore form the basis for further trials testing clinical benefits for app-surveyed HNC patients.

### Conclusions

The present trial aims to demonstrate the feasibility of ePROM monitoring of HNC patients during (chemo)radiation therapy. The planned secondary endpoints of this study will provide useful data regarding patterns of symptoms during therapy that may predict inferior outcomes or the occurrence of higher-grade treatment-related toxicities. In this respect, this trial will form the basis of future, larger investigations that will assess the potential clinical benefits of app-based monitoring for HNC patients.

## Appendix

Multimedia Appendix 1 SPIRIT chart for the trial.

## Abbreviations

EORTC: European Organisation for Research and Treatment of Cancer
ePROM: electronic patient-reported outcome measure
HNC: head and neck cancer
PSQ-18: Patient Satisfaction Questionnaire Short Form
SPIRIT: Standard Protocol Items: Recommendations for Interventional Trials
